# Capsule Endoscopy in Children

**DOI:** 10.3389/fped.2021.664722

**Published:** 2021-08-13

**Authors:** Stanley A. Cohen, Salvatore Oliva

**Affiliations:** ^1^Children's Center for Digestive Health Care, Atlanta, GA, United States; ^2^Pediatric Gastroenterology and Liver Unit, Maternal and Child Health Department, University Hospital Umberto I, Sapienza - University of Rome, Rome, Italy

**Keywords:** capsule endoscopy, pan-enteric capsule endoscopy, Crohn's disease, inflammatory bowel disease, small intestine, occult intestinal bleeding, capsule retention

## Abstract

Since its clearance for use throughout the world, capsule endoscopy (CE) has become an important diagnostic tool, helping us to understand and document both normal and abnormal findings in the small intestine, especially in children, since CE usually can be employed without sedation or radiation. The indications in children and adults are similar, though their relative frequencies are different, with evaluation of potential and known inflammatory bowel disease the most common in the pediatric population, with CE also yielding increased diagnostic certainty compared to radiographic studies and surrogate biomarkers. Newer capsules now create opportunities to expand that understanding and our practices so that we can learn when and how to employ CE and pan-enteric CE to better monitor and guide therapy. It will take further studies to determine the best uses for CE and how to select the appropriate candidates, especially with ongoing concern about capsule ingestion vs. placement, the potential for capsule retention (particularly in known Crohn's disease), still elusive optimal methods for bowel cleansing, and the most meaningful scoring for research and clinical use.

## Introduction

Consider that a swallowable video capsule, based on miniaturization technology applied to its electronic components, allows us to visualize and photograph the entire small intestine (1). That seemed like pure science fiction, based on old movie scripts, until the beginning of the twenty-first century, when the fantasy turned into a logical, startling reality. Introduced in 2001, the pill camera (Given Imaging, Yoqneam, Israel) received North American and European marketing clearance for patients of 10 years of age and older in 2003, and expanded to 2 years of age and older in 2009, with patency capsule use approved the same year, expanding the possibility of wide pediatric use (2).

Upgrading CE's technical aspects (dual or rotational cameras, wider field of vision, longer battery life), the software (dynamic imaging speed, real-time viewing), and better bowel cleansing have all improved diagnostic accuracy. However, these features differ on the six currently available CE systems that are available internationally (PillCam, Medtronic, formerly Given, US; Endoscapsule, Olympus, Japan; Mirocam, Intromedic, Korea; CapsoCam, Capso Vision, US; NaviCam, Ankon Technologies, China; and OMOM, Chongqing, China), though not all are available in every country.

The ability to visualize the small intestine, the only portion of the gastrointestinal tract previously outside the visual limits of traditional endoscopy, was particularly appealing because capsule endoscopy (CE) can usually be performed without anesthesia or radiation and discomfort of other imaging procedures. Those same benefits, as well as CE's sensitivity, drove a desire to make CE a less invasive, initial diagnostic study and one to monitor the mucosa both in the small intestine and beyond. A slightly larger colon capsule (Medtronic) and a pan-enteric capsule (dubbed the Crohn's capsule, Medtronic), to evaluate the small and large intestine in the same procedure, are already available in Europe. Additionally, an esophageal device (PillCam ESO2) was developed to evaluate Barrett's esophagus, but has found little use. With scant pediatric data available on the esophageal, colon, and Crohn's capsules, this review focuses on small intestinal CE and the newly emerging use of pan-enteric CE.

## Indications

American and European endoscopic societies have promulgated guidelines on the indications for CE (3–5). While both recommend CE for evaluation of obscure gastrointestinal bleeding and anemia (OGIBA) and suspicion of Crohn's disease (CD), they also suggest doing so after negative gastroscopy and ileocolonoscopy.

Of note, the relative frequency of those indications differs substantially in adults and children, and even within the pediatric population when stratified by age. OGIBA in adults accounts for 66% of the indications for CE, with evaluation of CD accounting for 10%, and 11% of CE performed for clinical symptoms of pain, diarrhea, and /or weight loss (6). According to a pediatric meta-analysis, the evaluation of suspected or known small intestinal CD is the most common pediatric indication for CE in children, accounting for 63% of the total (7). Over half of the procedures for inflammatory bowel disease (IBD) indications, and 44% of the total, relate to suspicion of CD, while 16% of the total CE were to monitor those with known CD (16% of total). The evaluation of abdominal pain, particularly in combination with diarrhea represents another 10% of the procedures (8–28) (Table 1).

**Table 1:** Indications, outcomes, and adverse events in capsule endoscopy procedures on pediatric and adult patients (8).

**Indications (%)**	**Pediatric**	**Under 8 years of age**	**Adult**
Bleeding and / or anemia	15	36	66
Inflammatory bowel disease	63	24	10
Abdominal pain	10	14	11
Polyps / neoplasms	8	-	3
Other	4	25	10
Positive findings (%)	61	67	59
Adverse events (%)			
Retained capsule	2.6	0.5	1.4
Incomplete procedures	13	7	16
Other	0.9	-	1.1

However, these clinical indications vary with age (25). Among children aged 1.5–7.9 years who underwent CE, OGIB, accounted for 30 (36%) of the 83 patients in the cohort. Suspected CD was the indication for 20 patients (24%) of CEs with 11 (55%) having positive findings; while three patients had CE to monitor their CD. Evaluation of abdominal pain, malabsorption, and protein loss each prompted CE for 12, 12, and nine patients (14, 14, and 11%), respectively; those with suspected CD or recurrent abdominal pain are typically older than those with protein losing enteropathy and / or malabsorption. In contrast, OGIB and CD in older children and teens accounts for only 13–24 and 40–86%, respectively, of the indications in those of 10–18 years of age (29).

### Polyposis

**Figure 1:**
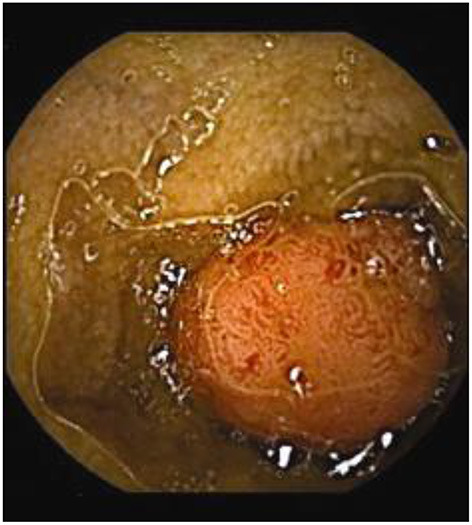
Polyp in the jejunum.

Assessment of polyposis syndromes in the SB demonstrates positive findings in 80.2% of CE in children, the highest diagnostic yield of any indication (18, 26). Considered “feasible, safe, and accurate” for the detection of small bowel polyps (Figure 1), CE allows for screening and surveillance of Peutz-Jeghers (PJS) and similar syndromes (familial adenomatous polyposis, Gardner's syndrome). While clinical guidelines generally recommend beginning to screen asymptomatic symptoms in those with PJS at 8 years of age, the frequency of repeating the exams every 1–5 years thereafter, and whether to do so with CE and then obtain an MRE or to directly proceed with deep enteroscopy for management are still debated (CE can miss proximal polyps, but CE and MRE are less invasive and together detect large and small polyps with accuracy equal to enteroscopy) (29, 30). Of note, screening the SB in cases of juvenile polyposis has shown no benefit (31).

### Inflammatory Bowel Disease

**Figure 2:**
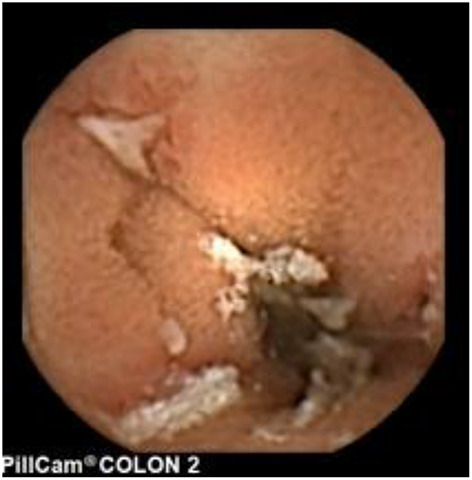
Moderate Crohn's with superficial and deepening ulcers.

**Figure 3:**
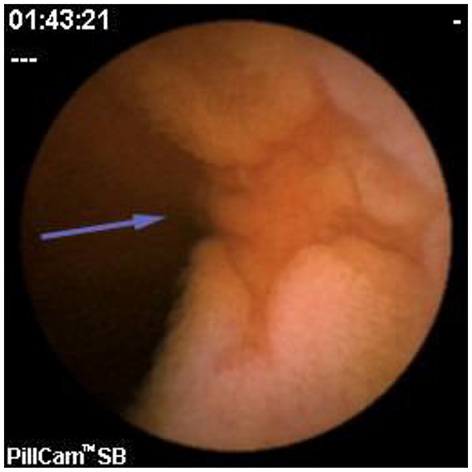
Deep erosion (presumably Crohn's).

**Figure 4:**
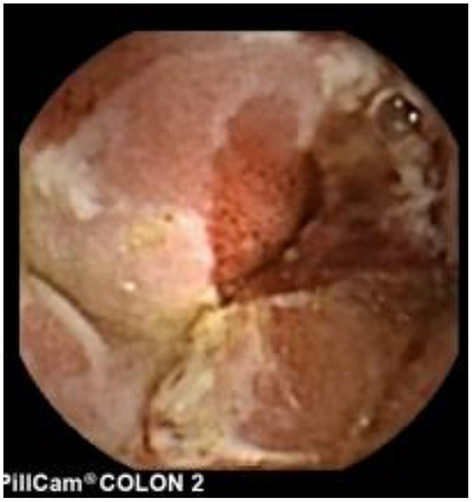
Severe Crohn's with ulceration and stenosis.

**Figure 5:**
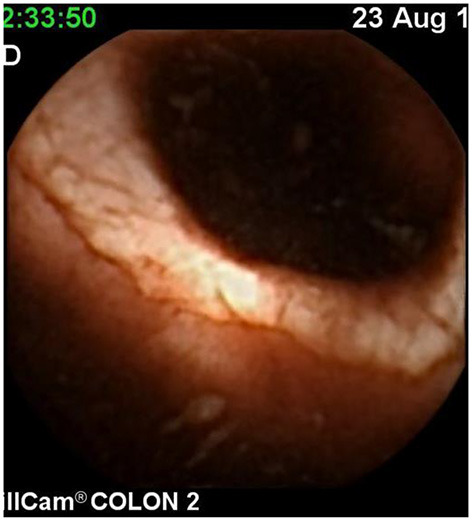
Crohn's small bowel stricture.

**Figure 6:**
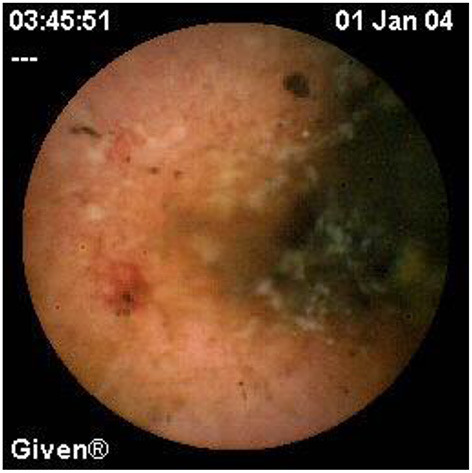
UC-like mucosa in jejunum (of a girl with prior colectomy).

**Figure 7:**
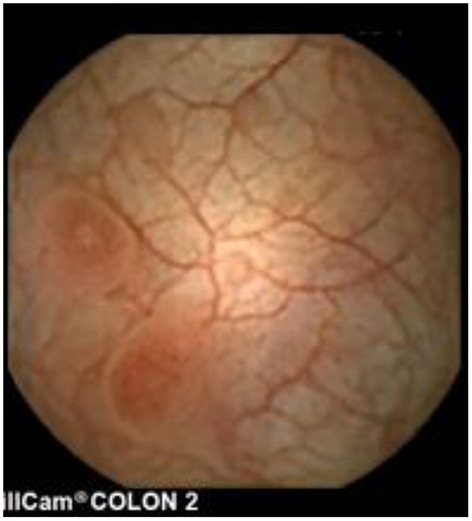
Apthous ulcers in the colon.

Since pediatric patients with CD will have small bowel (SB) involvement up to 70% of the time, with 40% estimated to have active disease exclusively in the SB, guidelines of European and North American societies suggest full evaluation of the gastrointestinal tract at the approximate time of CD diagnosis in pediatric patients to assess the extent/severity of CD and to clarify a classification of indeterminate colitis (30, 32–34) (Figures 2–7).

Repeated studies have shown the superiority of CE to accomplish that task, especially early onset or more proximal SB disease, either alone or following magnetic resonance imaging with oral contrast (MR enterography, MRE), which also can also detect strictures that would be a contraindication for CE (35–40).

A number of studies have now demonstrated the feasibility of sequential CE as a minimally invasive method to evaluate the mucosal response to treatment (41–45). Subsequently, pan-enteric capsule endoscopy (PCE) has been adapted to guide a treat-to-target therapeutic modifications strategy using a modified colon capsule to perform pan-enteric capsule endoscopy (PCE). In a cohort of 48 pediatric patients with CD, inflammation was present in 34 (71%) patients at baseline, 22 (46%) patients at 24 weeks, and 18 (39%) patients at 52 weeks (*P* < 0.05) (46). These findings resulted in therapeutic adjustment for 34 (71%) patients at baseline and 11 (23%) patients at 24 weeks based on PCE, while only 2 (4%) patients with PCE-negative results changed therapy based on their imaging studies. The treat-to-target strategy increased mucosal healing (MH) and deep remission (clinical and mucosal normality; DR) from 21% at baseline to 54% at 24 weeks and 58% at 52 weeks (*P* < 0.05 compared to baseline); whereas two patients (4%) did not respond to treatment.

Using an ITT analysis, complete MH at 52 weeks was associated with a decreased relapse rate clinically (*p* < 0.003), reduced steroid use (*p* < 0.0005), less treatment escalation (*p* < 0.0003), and decreased hospitalization (*p* < 0.0001). The decreased need for surgery was not statistically significant (*p* = 0.065). From the initial cohort, PCE was performed on 42 patients at 104 weeks (two developed an ileo-cecal valve stricture at 52 weeks; four were lost to follow-up) (47). MH decreased by 7% compared to their year 1 results.

At each assessment, PCE was compared to the other tested modalities. At 52 weeks, PCE showed DR in 28 (58%), complete MH in 6 (who had partial MH at 24 weeks), and new lesions detected in four subjects. MRE and SICUS had good concordance in evaluating DR (24/28, 86%), but they did not identify the new lesions in the four patients or mucosal improvements after therapy (*p* < 0.05). C-reactive protein and fecal calprotectin were not able to evaluate DR as well at 24 or 52 weeks (BR in 65 and 69%, respectively). The overall diagnostic yield of PCE, MRE, and biomarkers were 54, 37, and 33%, respectively (*p* < 0.05) (46).

However, to make these advances more effective, a challenge remains: to standardize CE interpretation in order to consistently diagnose and monitor CD findings. Two main CE scores exist for CD: the Lewis score (LS) and the CE Crohn's Disease Activity Index (CECDAI) (47, 48). While the LS is currently the most widespread CE score, the score is largely driven by stenosis and also includes villous edema, which is not considered a major feature of CD and it leads to the risk of errors in the assessment of mucosal healing (MH). Both indices have been used in small pediatric series, but remarkable discrepancies between the two were reported, with CECDAI better reflecting intestinal inflammation than LS (49). A new method, the Capsule Endoscopy - Crohn's Disease (CE-CD) index was devised adapting the Simple Endoscopic Score for Crohn's Disease (SES-CD), which is well-validated and widely used for ileocolonoscopy (50). Similar to SES-CD, CE-CD considers ulcers as elemental lesions of CD and takes into account the number of ulcers, size of the largest ulcer, the affected surface (as a percentage), and the presence or absence of stenosis in both the small and large intestine (Table 2). To date, the CE-CD has proven to be simple, reliable, and reproducible in the evaluation of SB inflammation in 312 pediatric patients with CD. This score seems also predictive of disease outcomes over time. The Pediatric Crohn's Disease Activity Index (PCDAI) appears to be correlated reasonably well (CE-CD ≥ 9; the area under the curve or AUC: 0.779) with a high specificity (90.1% for PCDAI ≥ 15) and low sensitivity (60.5%). Of particular note, 35 out 132 (26.5%) patients in clinical remission (PCDAI < 10) had surprisingly severe endoscopic patterns (CE-CD > 13), suggesting that CE-CD might be a useful pre-clinical predictor of CD exacerbations rather than overestimating disease severity (52).

**Table 2:** Simple endoscopic score for Crohn's disease (CE-CD) (51).

**Variable**	**0**	**1**	**2**	**3**
Size of ulcers	None	Aphthous ulcers (0.1–0.5 cm)	Large ulcers (0.5–2 cm)	Very large ulcers (>2 cm)
Ulcerated surface	None	<10%	10–30%	>30%
Affected surface	Unaffected segment	<50%	50–75%	>75%
Presence of narrowing (stenosis)	None	Single, can be passed	Multiple, can be passed	Cannot be passed

### Symptom-Based Evaluation

**Figure 8:**
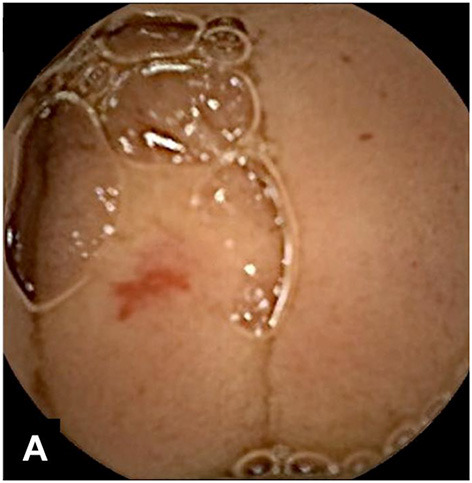
Arteriovenous malformation.

**Figure 9:**
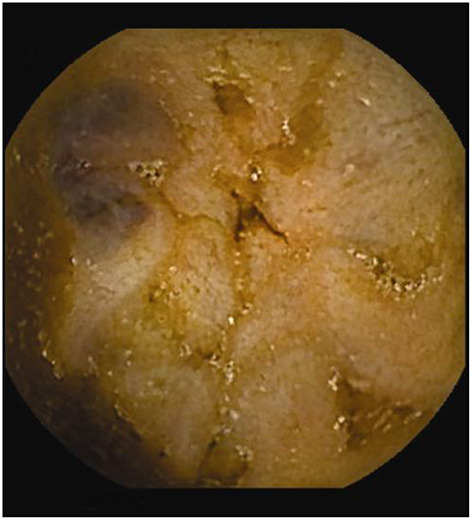
Vascular malformation.

**Figure 10:**
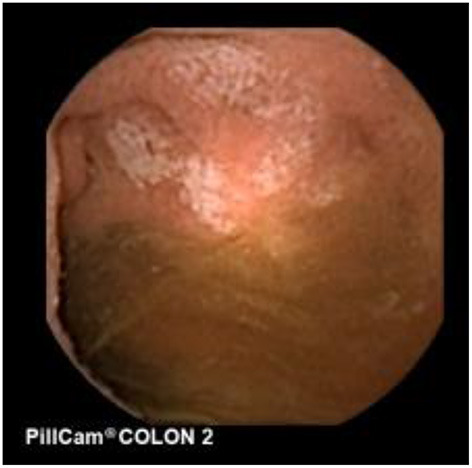
Lymphangiectasia (patchy).

**Figure 11:**
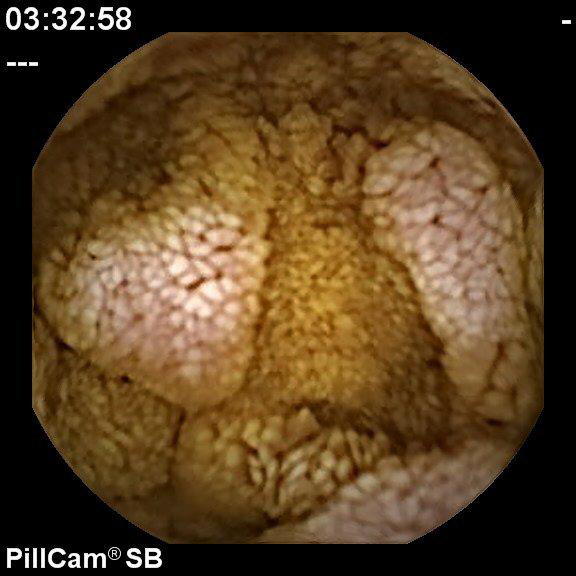
Lymphangiectasia (extensive).

**Figure 12:**
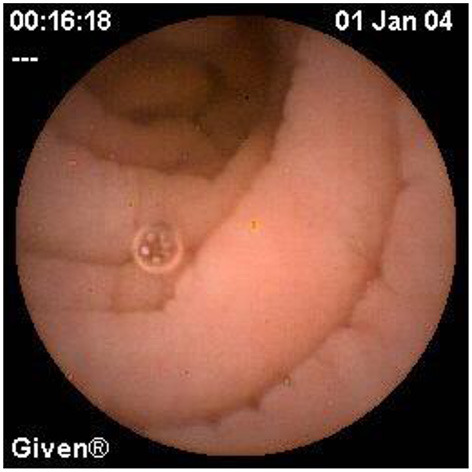
Celiac scalloping distally (with normal EGD).

**Figure 13:**
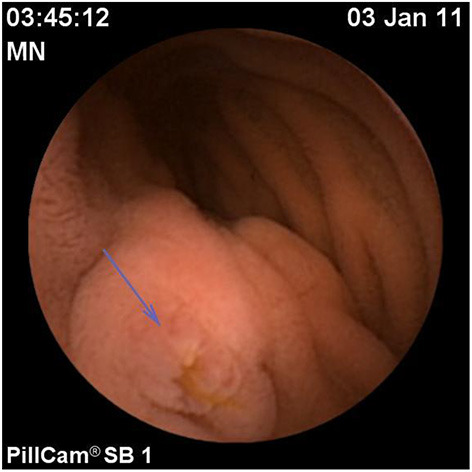
Ampulla of vater.

**Figure 14:**
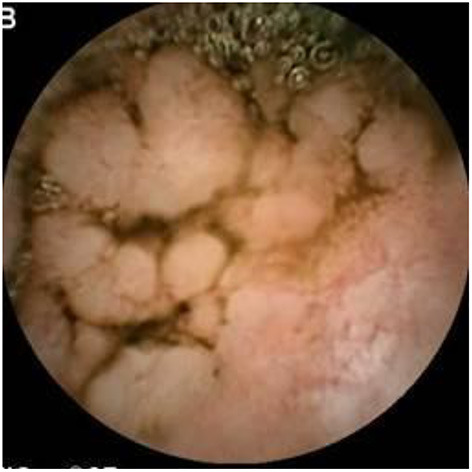
Lymphoid hyperplasia.

**Figure 15:**
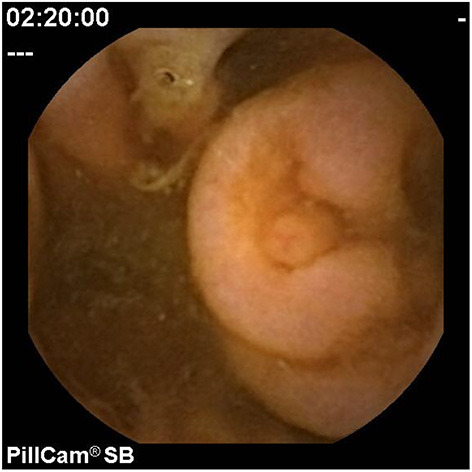
Intussusception.

In children, the diagnostic yield of CE for evaluation of OGIB is estimated to be 42% (7). In a study of 72 patients, positive findings in the assessment of abdominal pain with negative inflammatory markers were apparent in 21%, rising to 67% when inflammatory markers were present (51). However, the range of positive findings includes angioectasia and other vascular lesions (Figures 8, 9), Crohn's disease or other ulcers, gastritis, eosinophilic or other gastroenteropathy, polyps, graft-vs.-host disease, lymphangiectasia (Figures 10, 11), Meckel's diverticuli, scalloping or villous atrophy typical of celiac disease (Figure 12), and active bleeding without any source (29). Of note as well, CE has been used to acutely evaluate and re-evaluate graft-vs.-host disease after stem cell transplantation, and other protein-losing enteropathies. However, it also important to recognize that some findings are entirely normal (Figures 13–15).

### Pan-Enteric Capsule Endoscopy

The development of a slightly larger colon capsule (11.6 × 31.5 mm) with a 12-h battery life, two cameras with wider angles enabling nearly 360°, with a second iteration with higher resolution imaging of greater magnification than the first, and an “adaptive image acquisition rate” depending on the capsule's speed (53) has been able to be adapted and released in Europe, where it is termed a Crohn's capsule (Medtronic) PCE to evaluate both the small and large intestine in a single procedure.

While it has a few disadvantages: its larger size (though the same size as the colon capsule); bowel cleansing resembles that for a colonoscopy, with an additional booster dose during the actual procedure; and the procedure and reading times are longer, its utility has been shown in several studies.

The first published study evaluated the adapted devise in 40 pediatric subjects (age 13.1 ± 3.1 years) with known CD who underwent protocolized, comparative procedures in the course of disease re-evaluation. PCE demonstrated 90% sensitivity, 94% specificity in the SB, with PPV and NPV of 95 and 90%, respectively. PCE sensitivity was 89% in detecting colonic inflammation, while specificity was 100%. The positive predictive value (PPV) was 100% and negative predictive value (NPV) was 91% for colonic inflammation compared to MRE (sensitivity 85%, specificity 89%) and small intestine contrast ultrasonography (SICUS) (sensitivity 90%, specificity 83%). There were no serious adverse events related to the PCE procedure or the preparation reported (54).

Subsequently, the commercially available PCE and ileocolonoscopy (IC) were studied in 66 adult subjects with known CD and bowel patency. The diagnostic yield for active CD lesions was 83.3% for PCE and 69.7% for IC [95% confidence interval (CI), 2.6–24.7%]; with both modalities identifying active CD lesions in 65% of subjects. Of the 12 subjects where only PCE showed active CD, five had their lesions in the terminal ileum. Of note, IC, but not PCE, demonstrated active CD in three subjects (55). Two other larger studies of 99 and 93 adult patients subsequently reached similar conclusions, also showing the superiority of PCE over MRE, the latter study finding that C-reactive protein and fecal calprotectin were insensitive in recognizing active CD (0.48 and 0.59, respectively) (56, 57).

## Overcoming Capsule Issues

As with any procedure, even a minimally invasive one like CE, some capsule issues continue to present challenges, especially in children (28).

- Swallowing the capsule
- Endoscopic placement
- Capsule retention
- Bowel cleansing

### Swallowing the Capsule/Placement for Those Who Cannot Swallow

Swallowing the capsule may be difficult for some patients at any age (in the same way that some individuals are unable or unwilling to ingest pills). A technique called *stimulus fading* has been used to teach swallowing small, then progressively larger gelatin capsules or candies, with water, other liquids or even a small amount of yogurt, pudding, or apple sauce (58).

For those unable or unwilling to swallow a capsule, those with motility disorders or a tight esophageal sphincter, a capsule can be placed directly into the stomach, or preferably, the duodenum, during an endoscopy. This should be performed under general anesthesia, since capsules have been placed in the trachea when deep sedation was used (18). A front-loading capsule delivery devise (AdvanCE TM, US Endoscopy) can be used for older SB2 capsules. However, the newer SB3 and PCE capsules have cameras at each end, so that launching them with the extruder that pushes them out may mar the lens cover, interfering with interpretation. The alternative, a Roth Net (US Endoscopy), an extrudable fabric basket, has been shown to cause 50% more mucosal trauma, and may be difficult to use to launch a capsule in the small intestine (25).

A pediatric study compared the success rates and the differences in 51 CEs that were swallowed and 53 where it was placed. The median age was 12.8 (range: 1.6–18.5) years. Endoscopic placement was needed for children who were significantly younger (9.8 vs. 14.2 years; *P* < 0.001), lighter (34.5 vs. 54.9 kg; *P* < 0.0001), and had a longer small intestinal transit time (308 vs. 229 min; *P* < 0.0001). Children who ingested the capsule were more likely to have positive findings (50 vs. 30%, *P* = 0.017). Biopsies at the time of the endoscopy resulted in Iatrogenic bleeding and decreased visibility in 30% (16/53) of those who had CE placement, but that was not thought to change the outcome or subsequent patient management (59).

### Capsule Retention

Of note, prokinetics have made CE possible for those with esophageal or gastric motility disorders; however, intestinal dysmotility remains a contraindication similar to known stenosis or obstruction of the gastrointestinal tract, which would be most common in those with CD, those who have had intestinal resection, or undergone radiation to the abdomen. Furthermore, clinical signs of obstruction are a contraindication unless the passage of a self-dissolving patency capsule within timed guidelines (discussed below) or radiographic evidence proves that patency or surgery is considered a pre-procedure, as above. The potential for retention can also be discussed as being potentially therapeutic, in that it may identify a stricture. In at least one case, CE was performed specifically to help the surgeon identify the stricture intraoperatively (8).

A meta-analysis of 1,013 pediatric CE procedures documented gastric retention in four and SB retention in 18, a pooled retention rate of 2.3% (95%CI: 1.5–3.4%) (7). Endoscopy removed five capsules, four from the stomach and one from an ileal pouch with 13 surgically retrieved, simultaneously mitigating the cause of the retention. In one case, a bowel cleanout at 22 days post-ingestion evacuated a retained capsule.

Retention rates in children were 1.2% (95%CI: 0.9–1.6%), 2.6% (95%CI: 1.6–3.9%), and 2.1% (95%CI: 0.7–4.3%) for evaluation of occult gastrointestinal bleeding (OGIB), CD, and neoplastic lesions, respectively, with a pooled rate of 1.4% (95%CI: 1.2–1.6%) (8). On a per-procedure basis, this pattern is similar in adults, where capsule retention occurs at rate of 1.4, 2.2, and 1.2% in evaluation of OGIB, CD, and polyps, respectively (6).

The greatest risk factors for capsule retention overall is known IBD (5.2% risk), with that increasing when a previous small bowel follow-through (SBFT) demonstrated small bowel CD (35.7% risk) or if a body mass index below the fifth percentile is combined with known IBD (43% risk). However, retention has occurred despite the absence of strictures on SBFT (17). Among four patients with CD where the capsule passage lasted > 5 days (with three continuing on to retention), age was significant (18.8 ± 0.9 vs. 14.6 ± 3.5), but not height or weight, compared to patients who did not experience retention (17). Thus, it appears that the risk of retention is dependent on the clinical indication, and higher risk in patients with suspected chronic small bowel obstruction (60). No perforations, aspirations, or small bowel obstructions have been reported in children though rare cases have been reported in adults.

In a recent meta-analysis of 35 papers with 4,219 adult and pediatric patients with CD, 3.32% suffered from retention [95% confidence interval (CI), 2.62–4.2%]: this broke down to 4.63% (95% CI, 3.42–6.25%) in established CD and 2.35% (95% CI, 1.31–4.19%) in suspected CD. Retention rates were 3.49% (95% CI, 2.73–4.46%) in adults and 1.64% (95% CI, 0.68–3.89%) in those <18 years of age. Retention risk in established CD was 3.4 times higher than suspected CD in adults, but no difference existed in pediatric retention risk for established CD compared with suspected CD. In established CD, retention decreased if a patency capsule (2.88%; 95% CI, 1.74–4.74%) was used or MR/CT enterography (2.32%; 95% CI, 0.87–6.03%) was performed (61).

The majority of retentions have occurred despite normal SB radiographic studies, while radiologically documented strictures do not preclude functional patency allowing CE performance. A patency capsule (PC), identical in size to the SB capsule with a radiofrequency identity tag, was developed to address these concerns. The currently available version has barium, lactose, and dual timer plugs that gradually dissolve and disintegrate after 30 h.

Both a retrospective (9) and a prospective study (62) have been performed in pediatric IBD undergoing CE after using the first iteration of the PC (which had a 40-h time limit). In the retrospective analysis, CE was performed successfully in all but one of the 19 patients where patency was established. The prospective trial of 10–16-year-olds who ingested the PC found that 15 of 18 excreted an intact PC (mean 34.5 h) without any PC or CE retentions or adverse events (62). CD was eventually diagnosed in all patients having PC transit of more than 40 h and in nine of 12 who passed the patency capsule in 40 h or less. There were no capsule retentions or adverse events. Thus, the PC can serve as a useful guide and may lessen the likelihood of CE retention, particularly in known CD where the risk of retention is greatest.

### Other Contraindications

In pregnant women, CE should be restricted to urgent cases where diagnosis cannot be postponed, since safety data are not available. There is still an existing contraindication by manufacturers that those with an implanted cardio-assistive device should not have CE performed, though theoretical and clinical evidence suggest that VCE can be performed safely. Of note, patients undergoing an MRI with a capsule in the abdomen show susceptibility artifacts on their scans but show no evidence of clinical harm (63).

### Bowel Cleansing

Because of the inability to flush or suction fluids or gas during CE, adequate bowel cleaning is essential. Debris, bubbles, bile, and blood, particularly in the distal small bowel, limit CE's diagnostic ability (64). Various cleansing regimens have been tested in adults (65, 66). The only pediatric prospective study evaluated 198 patients with five different preparations (67). Of these, polyethylene glycol (PEG) solution, 1.75 g/25 mL per kg (up to 70 g/1,000 mL) the night prior the procedure with 20 mL (376 mg) of oral simethicone given 30 min was the most successful, lessening discomfort and improving visualization significantly in the distal ileum, the portion most often impaired by debris.

A specific score to evaluate cleansing in the SB for CE has recently been developed and validated by 20 readers who independently read 1,233 duplicate images 4 weeks apart. Each image was scored on two parameters: visualized mucosa and the degree of the image obscured by debris, bubbles, and bile. Almost perfect inter-rater and intra-rater reliability was observed for what is to be known as the KODA score and can be used for clinical trials (68).

A similar effort has been occurring for colon capsule cleansing. In that grading scale (CC-CLEAR), the colon is divided into three segments: right, transverse, and left colon. Each is classified by an estimation of the mucosa visualized clearly with the overall cleansing classification a sum of the segment scores, grading between inappropriate and excellent, although an inappropriate classification in any segment renders the entire score as inappropriate. That scale was considered superior to a previously developed score, the Leighton scale, on 58 consecutive colon capsules, with excellent inter and intra observer agreement (69).

The regimen devised for pediatric pan-enteric cleansing is based on what was used for the treat-to-target studies, getting an adequate cleaning level in >80% of cases (46, 47). This regimen primarily uses polyethylene glycol (PEG), includes domperidone, though metoclopramide can be substituted, and sodium phosphate (NaP) as a booster to speed up the capsule during the exam. This scheme was able to obtain completion and excretion rates higher than 95 and 84%, respectively.

## Conclusion

Over the two decades since its inception, CE has become part of our diagnostic armamentarium, helping us to understand and document both normal and abnormal findings in the small intestine. Newer capsules now create opportunities to expand that understanding and our practices so that we can learn when and how to employ CE and PCE to better monitor and guide therapy. This will take further studies to determine the best uses for CE and how to select the appropriate candidates.

## Author Contributions

Both authors listed have made a substantial, direct and intellectual contribution to the work, and approved it for publication.

## Conflict of Interest

SC and SO have served as consultants and have received research grants from Medtronics.

## Publisher's Note

All claims expressed in this article are solely those of the authors and do not necessarily represent those of their affiliated organizations, or those of the publisher, the editors and the reviewers. Any product that may be evaluated in this article, or claim that may be made by its manufacturer, is not guaranteed or endorsed by the publisher.

## References

[1] Meron GD. The development of the swallowable video capsule (M2A). Gastrointest Endosc. (2000) 52:817–9. 10.1067/mge.2000.110204 11115933

[2] U.S. Food and Drug Administration Center for Devices and Radiological Health. PC Patency System and Pillcam Platform with Pillcam SB Capsules. (2009). Available online at: http://www.accessdata.fda.gov/cdrh_docs/pdf9/K090557.pdf (accessed September 28, 2009).

[3] Mishkin DS Chuttani R Croffie J Disario J Liu J Shah R. ASGE Technology Status Evaluation Report: wireless capsule endoscopy. Gastro Endosc. (2006) 63:539–45. 10.1016/j.gie.2006.01.014 16564850

[4] Pennazio M Spada C Eliakim R Keuchel M May A Mulder CJ. Small-bowel capsule endoscopy and device-assisted enteroscopy for diagnosis and treatment of small-bowel disorders: European Society of Gastrointestinal Endoscopy (ESGE) Clinical Guideline. Endoscopy. (2015) 47:352–76. 10.1055/s-0034-1391855 25826168

[5] Escher M. Small intestinal capsule endoscopy: recommendations and limitations of the new ESGE-guideline 2015. Dtsch Med Wochenschr. (2015) 13:140. 10.1055/s-0041-102450

[6] Liao Z Gao R Xu C Zhao-Shen L. Indications and detection, completion, and retention rates of small-bowel capsule endoscopy: a systematic review. Gastrointest Endosc. (2010) 71:280–6. 10.1016/j.gie.2009.09.031 20152309

[7] Cohen SA. The potential applications of capsule endoscopy in pediatric compared to adult patients. Gastroenterol Hepatol. (2013) 9:92–7. 23983653 PMC3754776

[8] Cohen SA Klevens AI. Use of capsule endoscopy in diagnosis and management of pediatric patients, based on meta-analysis. Clin Gastroenterol Hepatol. (2011) 9:490–6. 10.1016/j.cgh.2011.03.025 21440674

[9] Cohen SA Ephrath H Lewis JD Klevens A Bergwerk A Liu S. Pediatric capsule endoscopy: a single center, 5 year retrospective review of small bowel and patency capsules. JPGN. (2012) 54:409–13. 10.1097/MPG.0b013e31822c81fd 21760541

[10] Gralnek IM Cohen SA Ephrath H. Small bowel capsule endoscopy impacts diagnosis and management of pediatric inflammatory bowel disease: a prospective study. Digest Dis Scien. (2012) 57:465–71. 10.1007/s10620-011-1894-5 21901253

[11] Tokuhara D Watanabe K Okano Y Tada A Yamato K Mochizuki T. Wireless capsule endoscopy in pediatric patients: the first series from Japan. J Gastroenterol. (2010) 45:683–91. 10.1007/s00535-010-0209-5 20143103

[12] Guilhon de. Araujo Sant'Anna AM, Dubois J, Miron MC, Seidman EG. Wireless capsule endoscopy for obscure small-bowel disorders: final results of the first pediatric controlled trial. Clin Gastroenterol Hepatol. (2005) 3:264–70. 10.1016/S1542-3565(04)00715-3 15765446

[13] Jensen MK Tipnis NA Bajorunaite R Sheth MK Sato TT Noel RJ. Capsule endoscopy performed across the pediatric age range: indications, incomplete studies, and utility in management of inflammatory bowel disease. Gastrointest Endosc. (2010) 72:95–102. 10.1016/j.gie.2010.01.016 20472231

[14] Antao B Bishop J Shawis R Thomson M. Clilnical application and diagnostic yield of wireless capsule endoscopy in children. J Laparoendosc Adv Surg Tech. (2007) 17:364–70. 10.1089/lap.2006.0114 17570790

[15] Cohen SA Gralnek IM Ephrath H Saripkin L Meyers W Sherrod O. Capsule endoscopy may reclassify pediatric inflammatory bowel disease: a historical analysis. J Pediatr Gastroenterol Nutr. (2008) 47:31–6. 10.1097/MPG.0b013e318160df85 18607266

[16] De' Angelis GL Fornaroli F de' Angelis N Magiteri B Bizzarri B. Wireless capsule endoscopy for pediatric small-bowel diseases. Am J Gastroenterol. (2007) 102:1749–57. 10.1111/j.1572-0241.2007.01209.x 17686071

[17] Atay O Mahajan L Kay M Mohr F Kaplan B Wyllie R. Risk of capsule endoscope retention in pediatric patients: a large single-center experience and review of the literature. J Pediatr Gastroenterol Nutr. (2009) 49:1–6. 10.1097/MPG.0b013e3181926b01 19561547

[18] Cohen S. Pediatric capsule endoscopy. Tech Gastrointest Endosc. (2013) 15:32–5. 10.1016/j.tgie.2012.09.002

[19] Moy L Levine J. Wireless capsule endoscopy in the pediatric age group: experience and complications. J Pediatr Gastroenterol Nutr. (2007) 44:516–20. 10.1097/MPG.0b013e3180335548 17414156

[20] Ge ZZ Chen HY Gao YJ Gu JL Hu YB Xiao SD. Clinical application of wireless capsule endoscopy in pediatric patients for suspected small bowel diseases. Eur J Pediatr. (2007) 166:825–9. 10.1007/s00431-006-0331-9 17103187

[21] Argüelles-Arias F Caunedo A Romero J Sánchez A Rodríguez-Téllez M Pellicer FJ. The value of capsule endoscopy in pediatric patients with a suspicion of Crohn's disease. Endoscopy. (2004) 36:869–73. 10.1055/s-2004-825854 15452782

[22] Urbain D Tresinie M De Looz D Demedts I Hauser B Mana F. Capsule endoscopy in paediatrics: multicentric Belgian study. Acta Gastroenterol Belg. (2007) 70:11–4. 17619532

[23] Barth BA Donovan K Fox VL. Endoscopic placement of the capsule endoscope in children. Gastrointest Endosc. (2004) 60:818–21. 10.1016/S0016-5107(04)02052-8 15557968

[24] Shamir R Hino B Hartman C Berkowitz D Eshach-Adiv O Eliakim R. Wireless video capsule in pediatric patients with functional abdominal pain. J Pediatr Gastroenterol Nutr. (2007) 44:45–50. 10.1097/01.mpg.0000239737.64240.72 17204952

[25] Fritscher-Ravens A Scherbakov P Bufler P Torroni F Ruuska T Nuutinen H. The feasibility of wireless capsule endoscopy in detecting small intestinal pathology in children under the age of 8 years: a multicentre European study. Gut. (2009) 58:1467–72. 10.1136/gut.2009.177774 19625281

[26] Postgate A Hyer W Phillips R Gupta A Burling D Bartram C. Feasibility of video capsule endoscopy in the management of children with Peutz-Jeghers Syndrome: a blinded comparison with barium enterography for the detection of small bowel polyps. J Pediatr Gastroenterol Nutr. (2009) 49:417–23. 10.1097/MPG.0b013e31818f0a1f 19543117

[27] Thomson M Fritscher-Ravens A Mylonaki M Swain P Eltumi M Heuschkel R. Wireless capsule endoscopy in children: a study to assess diagnostic yield in small bowel disease in pediatric patients. J Pediatr Gastroenterol Nutr. (2007) 44:192–7. 10.1097/01.mpg.0000252196.91707.ff 17255830

[28] Oliva S Cohen SA DiNardo G Gualdi G Cucchiara S Casciani E. Capsule endoscopy in pediatrics: a 10-years journey. World J Gastroenterol. (2014) 44:16603–8. 10.3748/wjg.v20.i44.16603 25469028 PMC4248203

[29] Zevit N Shamir R. Wireless capsule endoscopy of the small intestine in children. J Pediatr Gastroenterol Nutr. (2015) 60:696–701. 10.1097/MPG.0000000000000782 25782661

[30] Argurlles-Arias F Donat E Fernandez-Urien I Alberca F Arguelles-Martín F Martínez MJ. Guidelines for wireless capsule endoscopy in children and adolescents: a consensus document by the SEGHNP (Spanish Society for Pediatric Gastroenterology, Hepatology and Nutrition) and SEPD (Spanish Society for Digestive Diseases). Rev Esp Enferm Dig. (2015) 107:714–31. 10.17235/reed.2015.3921/2015 26671584

[31] Hoffman N Bass L. The utility of capsule endoscopy in children and adolescents with polyposis syndromes. NASPGHAN. (2020) 684.

[32] Levine A Koletzko S Turner D Escher JC Cucchiara S de Ridder L. ESPGHAN revised Porto criteria for the diagnosis of inflammatory bowel disease in children and adolescents. J Pediatr Gastroenterol Nutr. (2014) 58:795–806. 10.1097/MPG.0000000000000239 24231644

[33] Oliva S Thomson M de Ridder L. Martín-de-Carpi J Van Biervliet S Braegger C. Endoscopy in pediatric inflammatory bowel disease: a position paper on behalf of the Porto IBD group of the European Society for pediatric gastroenterology, hepatology and nutrition. J Pediatr Gastroenterol Nutr. (2018) 67:414–30. 10.1097/MPG.0000000000002092 30130311

[34] Crandall WV Boyle BM Colletti RB Margolis PA Kappelman MD. Development of process and outcome measures for improvement: lessons learned in a quality improvement collaborative for pediatric inflammatory bowel disease. Inflamm Bowel Dis. (2011) 17:2184–91. 10.1002/ibd.21702 21456033

[35] Solem CA Loftus EV Fletcher JG Baron TH Gostout CJ Petersen BT. Small-bowel imaging in Crohn's disease: a prospective, blinded, 4-way comparison trial. Gastrointest Endosc. (2008) 68:255–66. 10.1016/j.gie.2008.02.017 18513722

[36] Triester SL Leighton JA Leontiadis GI Gurudu SR Fleischer DE Hara AK. A meta-analysis of capsule endoscopy (CE) compared to other modalities in patients with non-stricturing small bowel Crohn disease. Am J Gastroenterol. (2006) 101:954–64. 10.1111/j.1572-0241.2006.00506.x 16696781

[37] Kopylov U Yung DE Engel T Vijayan S Har-Noy O Katz L. Diagnostic yield of capsule endoscopy versus magnetic resonance enterography and small bowel contrast ultrasound in the evaluation of small bowel Crohn's disease: systematic review and meta-analysis. Dig Liver Dis. (2017) 49:854–63. 10.1016/j.dld.2017.04.013 28512034

[38] González-Suárez B Rodriguez S Ricart E Ordás I Rimola J Díaz-González Á. Comparison of capsule endoscopy and magnetic resonance enterography for the assessment of small bowel lesions in Crohn's disease. Inflam Bowel Dis. (2018) 24:775–80. 10.1093/ibd/izx107 29506048 PMC6231365

[39] Aloi M Di Nardo G Romano G Casciani E Civitelli F Oliva S. Magnetic resonance enterography, small intestine contrast ultrasound, and capsule endoscopy to evaluate the small bowel in pediatric Crohn's disease: a prospective, blinded comparison study. Gastrointest Endosc. (2015) 81:420–7. 10.1016/j.gie.2014.07.009 25115363

[40] Hijaz NM Attard TM Colombo JM Mardis NJ Friesen CA. Comparison of the use of wireless capsule endoscopy with magnetic resonance enterography in children with inflammatory bowel disease. World J Gastroenterol. (2019) 25:3808–22. 10.3748/wjg.v25.i28.3808 31391775 PMC6676548

[41] Efthymiou A Viazis N Mantzaris G Papadimitriou N Tzourmakliotis D Raptis S. Does clinical response correlate with mucosal healing in patients with Crohn's disease of the small bowel? A prospective, case-series study using wireless capsule endoscopy. Inflamm Bowel Dis. (2008) 14:1542–7. 10.1002/ibd.20509 18521929

[42] Niv E Fishman S Kachman H Arnon R Dotan I. Sequential capsule endoscopy of the small bowel for follow-up of patients with known Crohn's disease. J Crohn's Colit. (2014) 8:1616–23. 10.1016/j.crohns.2014.03.003 24666976

[43] Hall BJ Holleran GE Smith SM Mahmud N McNamara DA A. prospective 12-week mucosal healing assessment of small bowel Crohn's disease as detected by capsule endoscopy. Eur J Gastroenterol Hepatol. (2014) 26:1253–9. 10.1097/MEG.0000000000000194 25264865

[44] Kopylov U Yablecovitch D Lahat A Neuman S Levhar N Greener T. Detection of small bowel mucosal healing and deep remission in patients with known small bowel Crohn's disease using biomarkers, capsule endoscopy, and imaging. Am J Gastroenterol. (2015) 110:1316–23. 10.1038/ajg.2015.221 26215531

[45] Melmed GY Dubinsky MC Rubin DT Fleisher M Pasha SF Sakuraba A. Utility of video capsule endoscopy for longitudinal monitoring of Crohn's disease activity in the small bowel: a prospective study. Gastrointest Endosc. (2018) 88:947–55. 10.1016/j.gie.2018.07.035 30086261

[46] Oliva S Aloi M Viola F Mallardo S Civitelli F Maccioni F. A treat to target strategy using panenteric capsule endoscopy in pediatric patients with Crohn's disease. Clin Gastroenterol Hepatol. (2019) 17:2060–7. 10.1016/j.cgh.2018.10.015 30326301

[47] Gralnek IM Defranchis R Seidman E Leighton JA Legnani P Lewis BS. Development of a capsule endoscopy scoring index for small bowel mucosal inflammatory change. Aliment Pharmacol Ther. (2008) 27:146–54. 10.1111/j.1365-2036.2007.03556.x 17956598

[48] Niv Y Ilani S Levi Z Hershkowitz M Niv E Fireman Z. Validation of the Capsule Endoscopy Crohn's Disease Activity Index (CECDAI or Niv score): a multicenter prospective study. Endoscopy. (2012) 44:21–6. 10.1055/s-0031-1291385 22125196

[49] Omori T Kambayashi H Murasugi S Ito A Yonezawa M Nakamura S. Comparison of Lewis score and capsule endoscopy Crohn's disease activity index in patients with Crohn's disease. Dig Dis Sci. (2020) 65:1180–8. 10.1007/s10620-019-05837-7 31541367

[50] Daperno M D'Haens G Van Assche G Baert F Bulois P Maunoury V. Development and validation of a new, simplified endoscopic activity score for Crohn's disease: the SES-CD. Gastrointest Endosc. (2004) 60:505–12. 10.1016/S0016-5107(04)01878-4 15472670

[51] Katsinelos P Fasoulas K Beltsis A Chatzimavroudis G Paroutoglou G Maris T. Diagnostic yield and clinical impact of wireless capsule endoscopy in patients with chronic abdominal pain with or without diarrhea: a Greek multicenter study. Eur J Intern Med. (2011) 22:e63–6. 10.1016/j.ejim.2011.06.012 21925046

[52] Oliva S Veraldi S Cucchiara S Spagnoli A Cohen SA. Assessment and validation of a new capsule endoscopy score for Crohn's disease (CE-CD) in children. Endosc Int Open. (In press). 10.1055/a-1522-8723 PMC8445685 34540539

[53] Hong SN Kang SH Jang HJ Wallace MB. Recent advance in colon capsule endoscopy: what's new? Clin Endosc. (2018) 51:334–43. 10.5946/ce.2018.121 30078307 PMC6078933

[54] Oliva S Cucchiara S Civitelli F Casciani E Di Nardo G Hassan C. Colon capsule endoscopy compared with other modalities in the evaluation of pediatric Crohn's disease of the small bowel and colon. Gastroint Endosc. (2016) 83:975–83. 10.1016/j.gie.2015.08.070 26363334

[55] Leighton JA Helper DJ Gralnek IM Dotan I Fernandez-Urien I Lahat A. Comparing diagnostic yield of a novel pan-enteric video capsule endoscope with ileocolonoscopy in patients with active Crohn's disease: a feasibility study. Gastroint Endosc. (2017) 85:196–205. 10.1016/j.gie.2016.09.009 27658907

[56] Bruining DH Oliva S Fleisher MR Fischer M Fletcher JG. Panenteric capsule endoscopy vs. ileocolonoscopy plus magnetic resonance enterography in Crohn's disease: a multicentre, prospective study. BMJ Gastro. (2019) 7:365. 10.1136/bmjgast-2019-000365 32499275 PMC7282309

[57] Tai FWD Ellul P Elosua A Fernandez-Urien I Tontini GE Elli L. Panenteric capsule endoscopy identifies proximal small bowel disease guiding upstaging and treatment intensification in Crohn's disease: a European multicentre observational cohort study. Unit Europ Gastroent J. (2020) 9:248–55. 10.1177/2050640620948664 32741315 PMC8259365

[58] Yoo JH Tarbox JJ Granpeesheh D. Using stimulus fading to teach a young child with autism to ingest wireless capsule endoscopy. Gastrointest Endosc. (2008) 67:1203–4. 10.1016/j.gie.2007.10.048 18249403

[59] Burgess CJ McIntyre EC Withers GD Ee LC. Comparing swallowing of capsule to endoscopic placement of capsule endoscopy in children. JGH Open J Gastroenterol Hepatol. (2017) 1:11–4. 10.1002/jgh3.12001 30483526 PMC6207005

[60] Singeap AM Trifan A Cojocariu C Sfarti C Stanciu C. Outcomes after symptomatic capsule retention in suspected small bowel obstruction. Eur J Gastroenterol Hepatol. (2011) 23:886–90. 10.1097/MEG.0b013e328349efa4 21811157

[61] Pasha SF Pennazio M Rondonotti E Wolf D Buras MR Albert JG. Capsule retention in Crohn's disease: a meta-analysis. Inflamm Bowel Dis. (2020) 26:33–42. 10.1093/ibd/izz083 31050736

[62] Cohen SA Gralnek IM Ephrath H Stallworth A Wakhisi T. The use of a patency capsule in pediatric Crohn's disease: a prospective evaluation. Dig Dis Sci. (2011) 56:860–5. 10.1007/s10620-010-1330-2 20652742

[63] Bandorski D Kurniawan N Baltes P Hoeltgen R Hecker M Stunder D. Contraindications for video capsule endoscopy. World J Gastroenterol. (2016) 22:9898–908. 10.3748/wjg.v22.i45.9898 28018097 PMC5143757

[64] Niv Y. Efficiency of bowel preparation for capsule endoscopy examination: a meta-analysis. World J Gastroenterol. (2008) 14:1313–7. 10.3748/wjg.14.1313 18322940 PMC2693674

[65] Rokkas T Papaxoinis K Triantafyllou K Pistiolas D Ladas SD. Does purgative preparation influence the diagnostic yield of small bowel video capsule endoscopy? a meta-analysis. Am J Gastroenterol. (2009) 104:219–27. 10.1038/ajg.2008.63 19098872

[66] Chen HB Huang Y Chen SY Song HW Li XL Dai DL. Small bowel preparations for capsule endoscopy with mannitol and simethicone: a prospective, randomized, clinical trial. J Clin Gastroenterol. (2011) 45:337–41. 10.1097/MCG.0b013e3181f0f3a3 20871410

[67] Oliva S Cucchiara S Spada C Hassan C Ferrari F Civitelli F. Small bowel cleansing for capsule endoscopy in paediatric patients: a prospective randomized single-blind study. Dig Liver Dis. (2014) 46:51–5. 10.1016/j.dld.2013.08.130 24041737

[68] Alageeli M Yan B Alshankiti S Al-Zahrani M Bahreini Z Dang TT. KODA score: an updated and validated bowel preparation scale for patients undergoing small bowel capsule endoscopy. Endosc Int Open. (2020) 8:E1011–7. 10.1055/a-1176-9889 32743051 PMC7373654

[69] de Sousa Magalhães R Arieira C Boal Carvalho P Rosa B Moreira MJ Cotter J. Colon Capsule Cleansing Assessment and Report (CC-CLEAR): a new approach for evaluation of the quality of bowel preparation in capsule colonoscopy. Gastrointest Endosc. (2020) 93:212–23. 10.1055/s-0040-1704854 32534054

